# Randomized clinical trial of high versus low inferior mesenteric artery ligation during anterior resection for rectal cancer

**DOI:** 10.1002/bjs5.71

**Published:** 2018-06-08

**Authors:** S. Fujii, A. Ishibe, M. Ota, K. Watanabe, J. Watanabe, C. Kunisaki, I. Endo

**Affiliations:** ^1^ Department of Gastroenterological Surgery, Ichikawa Hospital International University of Health and Welfare Chiba Japan; ^2^ Department of Surgery, Gastroenterological Centre Yokohama City University Yokohama Japan; ^3^ Department of Gastroenterological Surgery, Graduate School of Medicine Yokohama City University Yokohama Japan

## Abstract

**Background:**

The optimal level for inferior mesenteric artery ligation during anterior resection for rectal cancer is controversial. The aim of this randomized trial was to clarify whether the inferior mesenteric artery should be tied at the origin (high tie) or distal to the left colic artery (low tie).

**Methods:**

Patients were allocated randomly to undergo either high‐ or low‐tie ligation and were stratified by surgical approach (open or laparoscopic). The primary outcome was the incidence of anastomotic leakage. Secondary outcomes were duration of surgery, blood loss and 5‐year overall survival.

**Results:**

Some 331 patients entered the trial between June 2006 and September 2012. The trial was stopped prematurely as recruitment was slow. Seven patients were excluded after randomization but before operation because of procedural changes. High tie and low tie were performed in 164 and 160 patients respectively. The incidence of anastomotic leakage was not significantly different (17·7 versus 16·3 per cent respectively; P = 0·731). The incidence of severe complications requiring intervention was 2·4 versus 5·0 per cent for high and low tie respectively (P = 0·222). In multivariable analysis, risk factors for anastomotic leakage included male sex (odds ratio 4·36, 95 per cent c.i. 1·56 to 12·18) and distance of the tumour from the anal verge (odds ratio 0·99, 0·98 to 1·00). At 5 years there were no significant differences in overall (87·2 versus 89·4 per cent respectively; P = 0·386) and disease‐free (76·3 versus 77·6 per cent; P = 0·765) survival.

**Conclusion:**

The level of ligation of the inferior mesenteric artery does not significantly influence the rate of anastomotic leakage. Registration number: NCT01861678 ( https://clinicaltrials.gov).

## Introduction

In rectal cancer surgery the inferior mesenteric artery (IMA) can be ligated at its origin from the aorta (high tie) or distal to the branch of the left colic artery (LCA) (low tie). High‐tie ligation has been advocated^1^, ^2^, ^3^, ^4^, ^5^, ^6^ because it allows more radical resection and more accurate pathological staging. Others^7^, ^8^, ^9^, ^10^, ^11^, ^12^, ^13^ favour low‐tie ligation because of increased blood flow to the proximal end of the anastomosis. This debate goes all the way back to the descriptions by Miles^14^ and Moynihan^15^ in 1908. Recent studies^7^, ^8^, ^9^, ^10^ have recommended low tie, as there was no significant difference in survival rates between high‐ and low‐tie ligation.

Some^16^ have suggested that high tie should be restricted to patients with clinical suspicion of involved nodes around the origin of the IMA or to those who require additional vascular mobilization to construct a tension‐free anastomosis. Japanese guidelines^17^ recommend that upward lymph node dissection should be performed at the level of the IMA for clinical T2 or more advanced disease. There is no consensus, however, on where to divide the IMA. Several reviews^18^, ^19^, ^20^, ^21^ found no significant difference between high and low tie with regard to short‐ and long‐term results, with different authors recommending different methods. All emphasized the need for RCTs^18^ ^20^, ^21^. In the present study, patients with rectal cancer were randomized between high‐ and low‐tie ligation.

## Methods

This was a single‐centre phase III RCT, conducted at Yokohama City University Medical Centre. About 80 patients with rectal cancer were operated on annually at this institute. Patients with rectal cancer who were scheduled to undergo anterior resection were eligible for inclusion. All tumours were defined according to the seventh edition of the Japanese General Rules for Clinical and Pathological Studies on Cancer of the Colon, Rectum and Anus^22^. The rectum is defined as the intestine between the level of the sacral promontory and the upper edge of the puborectal muscle. The clinical TNM classification for the staging of rectal cancer was based on colonoscopy, CT of the thorax, abdomen and pelvis, abdominal ultrasonography or MRI. The general condition of all patients undergoing elective surgery was assessed before surgery by an anaesthetist.

Inclusion criteria were: age 20 years or above and histologically proven adenocarcinoma of the rectum. Exclusion criteria were: a primary tumour that directly invaded another organ clinically (T4b), synchronous distant or peritoneal metastasis, operation scheduled as an emergency, previous history of colorectal surgery except for appendicectomy, active or recent treatment for malignancy in another organ, and multiple colorectal cancers that needed construction of two or more anastomoses. Pregnant and lactating women were excluded, as were patients scheduled for resection without colorectal anastomosis.

Patients provided written informed consent. The trial protocol was approved by the ethics committee of Yokohama City University. The trial was registered at http://clinicaltrials.gov (trial number NCT01861678).

Patients were allocated randomly to undergo high‐ or low‐tie ligation of the IMA in a 1 : 1 ratio. Immediately before the operation, the surgeon in charge reported a registration to the Epidemiology Data Centre in the Department of Biostatistics, Yokohama City University, via the internal line of the hospital; randomization was done by an epidemiologist using the minimization method. To balance surgical backgrounds between high‐ and low‐tie groups, patients were stratified by surgical approach (open or laparoscopic). The study was conducted by the open‐label method. Blinding was not attempted.

### Interventions

All surgical procedures were performed by a specialized colorectal treatment team. The surgeon in charge of the team had acquired a specialist qualification from the Japan Society of Coloproctology^23^, which recognized his years of clinical experience in approved facilities and successful completion of the specialist qualifying examination^24^. Laparoscopic operations were performed by a surgeon who was similarly accredited by the Japanese Society for Endoscopic Surgery^25^. All operations were performed according to the standard procedure described in the seventh edition of the Japanese General Rules for Clinical and Pathological Studies on Cancer of the Colon, Rectum and Anus^22^.

For high‐tie ligation, the IMA was divided at its origin from the abdominal aorta. For low‐tie ligation, the IMA was divided just after branching to the LCA. Dissection of lymph nodes around the IMA was added in low‐tie ligation.

Conventional open surgery was performed in patients with bulky tumours (6 cm or larger). Other patients underwent laparoscopic surgery via a medial‐to‐lateral approach. The IMA was divided at the level according to allocation. Mobilization of the left colon was performed. The rectum distal to the tumour was divided with a linear stapler after rectal irrigation. Partial mesorectal excision was usually performed. The proximal colon was divided at least 10 cm from the lesion^22^. The distal margin was 3 cm for tumours above the peritoneal reflection and 2 cm for those in the mid and distal rectum^22^.

A haemorrhage test of the marginal artery was performed at the planned side of division. When the artery did not bleed, the colon was resected until bleeding was confirmed.

Reconstruction was undertaken using an end‐to‐end double stapling technique. An air leak test was done after reconstruction. For laparoscopic operations, an abdominal incision longer than 8 cm was considered a conversion.

In open surgery, the left colon was dissected from the retroperitoneum and mobilized. Lymph node dissection around the IMA was performed. A pelvic side‐wall lymphadenectomy was done for cT3–4 lower rectal cancers. As laparoscopic pelvic side‐wall lymphadenectomy was not conducted at the start of the study, this procedure was performed by an open approach. All other steps were as described for the laparoscopic approach. Creation of a diverting stoma was left to the surgeon in charge. An intraluminal drainage tube was inserted from the anus in the absence of a diverting stoma.

The time from skin incision to ligation of the IMA was recorded. Complications were graded according to the Clavien–Dindo classification^26^. Complications occurring within 30 days of surgery were considered as early, and those beyond this time as late.

### Pathology

Pathological results were recorded according to the seventh edition of the Japanese General Rules for Clinical and Pathological Studies on Cancer of the Colon, Rectum and Anus^22^ and the seventh edition for TNM classification^27^. Total numbers of harvested lymph nodes were counted, as well as for each lymph node station separately. Lymph node stations were divided as: the area of IMA origin; the intermediate region along the IMA; and the perirectal region around the marginal vessels. Pathological proximal and distal margins were recorded, and circumferential margin involvement was defined as exposure of a cancer cell at the dissection surface on histological examination.

### Neoadjuvant and adjuvant therapy, and follow‐up

Patients did not have neoadjuvant chemotherapy, radiotherapy or chemoradiotherapy. When the pathological stage was IIb, IIc or III by histological examination, adjuvant treatment with oral or intravenous fluoropyrimidine‐based chemotherapy was recommended.

The follow‐up schedule was based on tumour stage. For stages 0 (defined as Tis in the Japanese Classification of Colorectal Carcinoma; adenocarcinoma was detected at the mucosa layer on the histological examination in stage 0) and I, follow‐up included outpatient examinations with assessment of serum carcinoembryonic antigen (CEA), and chest, abdominal and pelvic CT once a year for 5 years. For stages II and IIIa, follow‐up included outpatient examinations with assessment of serum CEA, and chest, abdominal and pelvic CT every 6 months for the first 3 years and once‐yearly thereafter until 5 years after surgery. For stages IIIb and IIIc, follow‐up included outpatient examinations with assessment of serum CEA, and chest, abdominal and pelvic CT every 4 months for the first 2 years, and every 6 months thereafter until 5 years after surgery. For stage IV disease, which was seen occasionally after randomization, the follow‐up schedule was decided according to the condition of each patient.

### Primary and secondary outcome measures

Primary outcome was the rate of anastomotic leakage. Leakage was defined as an incontinuity at the anastomosis detected clinically or radiologically. Contrast radiography via the drainage tube was not done in all patients. However, contrast radiography was performed in patients with purulent discharge via an abdominal drainage tube, or peritonitis. If fistula was confirmed by contrast radiography, a patient was diagnosed as having an anastomotic leakage. Anastomotic leakage was categorized according to the Clavien–Dindo system^26^.

Secondary outcomes were duration of surgery, blood loss and 5‐year overall survival rate.

### Statistical analysis

It was hypothesized that low‐tie ligation would decrease the rate of anastomotic leakage from 15 to 6 per cent. Using a power of 80 per cent and α of 0·05, a sample size of 362 patients was needed. A dropout rate of approximately 10 per cent was anticipated. Therefore, 400 patients had to be included in this study. Enrolment was scheduled for 5 years after inclusion of the first patient.

Data were analysed according to the intention‐to‐treat principle. SAS^®^ software version 9.2 for Windows^®^ (SAS Institute, Cary, North Carolina, USA) was used for statistical analysis.

Categorical variables are presented as frequencies and percentages. Continuous variables are presented as mean(s.d.) values. The χ^2^ test and Student's *t* test were used to compare categorical and continuous variables respectively. Survival was analysed by the Kaplan–Meier method, and the difference between high‐ and low‐tie ligation was analysed with the log rank test. Risk factors for anastomotic leakage were assessed by logistic regression using a forward method. Variables with *P* < 0·100 were entered into multivariable analysis. *P* < 0·050 was considered statistically significant. All analyses were two‐sided.

## Results

**Figure 1:**
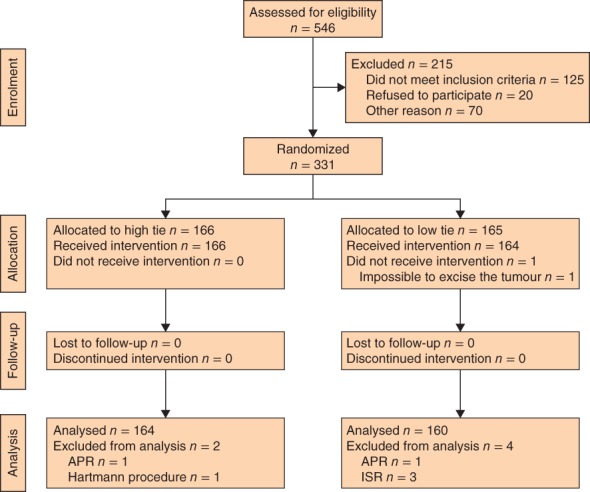
CONSORT diagram for the trial. For high‐tie ligation the inferior mesenteric artery was divided at its origin from the abdominal aorta; for low‐tie ligation the inferior mesenteric artery was divided just after branching to the left colic artery. APR, abdominoperineal resection of rectum; ISR, intersphincteric resection of rectum

Some 331 patients were randomized between June 2006 to September 2012 (*Fig*. 1). Due to slow recruitment, the trial was stopped prematurely. One hundred and sixty‐six patients were assigned to the high‐tie group and 165 to the low‐tie group. Two patients in the high‐tie group were excluded because of changes in operative procedure: one underwent an abdominoperineal rectal resection (APR) and the other had a Hartmann procedure. Five patients in the low‐tie group were excluded because of changes in operative procedure: three patients underwent an intersphincteric rectal resection, one had an APR, and one tumour could not be excised. In the low‐tie group, the LCA of one patient was separated during operation because of a high‐tension anastomosis. This patient was analysed in the low‐tie group according to allocation.

The primary outcome could be analysed for 164 patients in the high‐tie group and 160 in the low‐tie group. Patient and tumour characteristics are shown in *Table* 1. Second synchronous colorectal carcinomas were seen fairly frequently and mainly included Tis tumours within 10 cm of the rectal carcinoma necessitating additional distal sigmoid resections. Simultaneous resection of another organ was needed by six patients in the high‐tie group (hysterectomy, 3; oophorectomy, 1; partial resection of the jejunum, 2) and ten in the low‐tie group (hysterectomy, 4; oophorectomy, 2; partial resection of the ileum, 2; partial resection of the urinary bladder, 2).

**Table 1:** Patient and tumour characteristics

	High tie (*n* = 164)	Low tie (*n* = 160)
Age (years)*	65·9(10·4)	65·6(11·5)
Sex ratio (M : F)	103 : 61	97 : 63
ASA grade		
1	39 (23·8)	53 (33·1)
2	115 (70·1)	95 (59·4)
3	10 (6·1)	12 (7·5)
ECOG performance status		
0	72 (43·9)	78 (48·8)
1	77 (47·0)	61 (38·1)
2	15 (9·1)	21 (13·1)
Prognostic Nutrition Index*	52·3(6·9)	52·2(5·3)
Concomitant disease†	116 (70·7)	102 (63·8)
Second synchronous colonic cancer	15 (9·1)	18 (11·3)
Cardiovascular disease	79 (48·2)	71 (44·4)
Diabetes	19 (11·6)	30 (18·8)
Other	57 (34·8)	48 (30·0)
History of laparotomy	21 (12·8)	28 (17·5)
BMI (kg/m^2^)*	23·0(3·2)	22·4(3·5)
Tumour location		
Upper rectum	107 (65·2)	99 (61·9)
Lower rectum	57 (34·8)	61 (38·1)
Distance from anal verge (mm)*	88·7(32·9)	89·6(37·0)
Tumour diameter (mm)*	41·5(20·8)	41·9(20·5)
Histology		
Papillary adenocarcinoma	1 (0·6)	1 (0·6)
Well differentiated adenocarcinoma	81 (49·4)	85 (53·1)
Moderately differentiated adenocarcinoma	77 (47·0)	65 (40·6)
Poorly differentiated adenocarcinoma	1 (0·6)	2 (1·3)
Mucinous adenocarcinoma	3 (1·8)	2 (1·3)
Carcinoid tumour	1 (0·6)	4 (2·5)
Small cell carcinoma	0 (0)	1 (0·6)
pTNM stage		
0	4 (2·4)	6 (3·8)
1	56 (34·1)	54 (33·8)
2	43 (26·2)	36 (22·5)
3	54 (32·9)	56 (35·0)
4	7 (4·3)	8 (5·0)
Surgical approach		
Open	57 (34·8)	52 (32·5)
Laparoscopic	107 (65·2)	108 (67·5)
Level of anastomosis from anal verge (cm)*	5·8(2·0)	5·7(2·1)
Diverting stoma	36 (22·0)	47 (29·4)
Insertion of intraluminal drain from anus	12 (7·3)	19 (11·9)
Simultaneous resection of other organ	6 (3·7)	10 (6·3)
No. of linear stapler cartridges used		
1	120 (73·2)	111 (69·4)
≥ 2	44 (26·8)	49 (30·6)
Pelvic side‐wall lymphadenectomy	25 (15·2)	22 (13·8)
Adjuvant chemotherapy	39 (23·8)	46 (28·8)

Values in parentheses are percentages unless indicated otherwise;

* values are mean(s.d.).

† Some patients had more than one concomitant disease.

ECOG, Eastern Cooperative Oncology Group.

### Anastomotic leakage

The overall rate of anastomotic leakage was 17·7 per cent in the high‐tie group and 16·3 per cent in the low‐tie group (*P* = 0·731) (*Table* 2). For grade 2 or higher leakage the rate was 9·8 and 8·8 per cent respectively (*P* = 0·755), and for grade 3 or higher it was 2·4 and 5·0 per cent (*P* = 0·222). The 12 patients with grade 3 and 5 anastomotic leakage underwent reoperation with creation of a stoma. All but the one patient who died underwent stoma reversal later.

**Table 2:** Short‐term outcomes

	High tie (*n* = 164)	Low tie (*n* = 160)	*P* †
Anastomotic leakage			
All grades	29 (17·7)	26 (16·3)	0·731
Grade 2 or above	16 (9·8)	14 (8·8)	0·755
Grade 3 or above	4 (2·4)	8 (5·0)	0·222
Leakage grade			
1	13 (7·9)	12 (7·5)	0·432
2	12 (7·3)	6 (3·8)	
3	4 (2·4)	7 (4·4)	
4	0 (0)	0 (0)	
5	0 (0)	1 (0·6)	
Mortality	0 (0)	1 (0·6)	0·311
Early complication (except leakage)	61 (37·2)	56 (35·0)	0·681
Surgical‐site infection	8 (4·9)	10 (6·3)	0·590
Ileus	16 (9·8)	8 (5·0)	0·102
Enteritis	2 (1·2)	2 (1·3)	1·000
Chylous ascites	3 (1·8)	5 (3·1)	0·452
Urinary tract infection	1 (0·6)	2 (1·3)	0·547
Urinary dysfunction	4 (2·4)	3 (1·9)	0·727
Conversion to open surgery	6 of 107 (5·6)	2 of 108 (1·9)	0·142
Estimated blood loss (ml)*	155(299)	152(289)	0·867‡
Blood transfusion	3 (1·8)	3 (1·9)	0·976
Duration of surgery (min)*	209(67)	206(59)	0·672‡
Duration of IMA tie from start (min)*	41(15)	52(15)	< 0·001‡
Duration of laparoscopic procedure (min)*	161(42)	165(45)	0·525‡
Postoperative hospital stay (days)*	17(14)	16(12)	0·451‡

Values in parentheses are percentages unless indicated otherwise;

* values are mean(s.d.). IMA, inferior mesenteric artery.

† χ^2^ test, except

‡ Student's *t* test.

### Risk factors for anastomotic leakage

In univariable analysis, male sex, advanced T status (T4), transfusion of red blood cells, conversion to open surgery, distance of tumour from the anal verge and the number of stapler cartridges fired were associated significantly with the rate of anastomotic leakage. In multivariable analysis, male sex and distance of tumour from the anal verge were identified as independent risk factors for anastomotic leakage (*Table* 3).

**Table 3:** Risk factors for anastomotic leakage in all grades

	Leakage		Univariable analysis		Multivariable analysis	
	Yes (*n* = 55)	No (*n* = 269)	Odds ratio†	*P*	Odds ratio†	*P*
Sex						
M	47 (23·5)	153 (76·5)	4·40 (2·00, 9·67)	< 0·001	4·36 (1·56, 12·18)	0·005
F	8 (6·5)	116 (93·5)	1·00 (reference)		1·00 (reference)	
pT category						
pTis–pT3	39 (14·8)	225 (85·2)	1·00 (reference)		1·00 (reference)	
pT4	16 (27)	44 (73)	2·11 (1·08, 4·11)	0·028	1·57 (0·58, 4·24)	0·371
Blood transfusion						
Yes	4 (67)	2 (33)	10·28 (1·83, 57·59)		1·27 (0·06, 25·97)	0·875
No	51 (16·0)	267 (84·0)	1·00 (reference)	0·008	1·00 (reference)	
Conversion of laparoscopy						
Yes	4 (50)	4 (50)	5·09 (1·21, 21·34)		3·14 (0·69, 14·25)	0·139
No	34 (16·4)	173 (83·6)	1·00 (reference)	0·026	1·00 (reference)	
Distance of tumour from anal verge (mm)*	81(31)	91(35)	0·99 (0·98, 1·00)	0·043	0·99 (0·98, 1·00)	0·011
No. of stapler firings*	1·30(0·56)	1·49(0·64)	1·64 (1·05, 2·56)	0·031	1·09 (0·62, 1·91)	0·765

Values in parentheses are percentages unless indicated otherwise;

* values are mean(s.d.) and

† 95 per cent confidence intervals in parentheses.

### Surgical parameters, complications and pathology

Duration of surgery did not differ significantly between groups, although time to ligation of the IMA was significantly longer in the low‐tie group (*P* < 0·001) (*Table* 2). Blood loss did not differ significantly between the groups. The overall early complication rate was not significantly different, and neither was the total number of lymph nodes harvested or the number of lymph nodes per station. Proximal and distal pathological margins, and the positive circumferential margin rate were similar in high‐ and low‐tie groups (*Table* 4). All patients with positive IMA root nodes also had positive intermediate or perirectal lymph nodes.

**Table 4:** Oncological quality of surgery

	High tie (*n* = 164)	Low tie (*n* = 160)	*P* †
No. of lymph nodes harvested*			
Total	26·4(11·4)	24·1(12·2)	0·079‡
IMA root nodes	2·8(2·1)	2·9(2·7)	0·639‡
Intermediate lymph nodes	5·4(3·9)	5·1(3·9)	0·623‡
Perirectal lymph nodes	15·5(7·6)	14·1(7·5)	0·130‡
Lymph node involvement at each station			
IMA root nodes	3 (1·8)	5 (3·1)	0·452
Intermediate lymph nodes	10 (6·1)	8 (5·0)	0·666
Perirectal lymph nodes	58 (35·4)	55 (34·4)	0·852
IMA root nodes positive, intermediate nodes negative	0 (0)	1 (0·6)	0·311
Intermediate nodes positive, perirectal nodes negative	1 (0·6)	1 (0·6)	0·986
Pathological proximal margin (cm)*	13·4(5·5)	12·5(4·9)	0·110‡
Pathological distal margin (cm)*	3·1(1·8)	3·2(2·0)	0·618‡
Positive circumferential margin	3 (1·8)	5 (3·1)	0·452

* Values are mean(s.d.).

IMA, inferior mesenteric artery.

† χ^2^ test, except

‡ Student's *t* test.

### Long‐term results

The 5‐year overall survival rate did not differ significantly between high‐ and low‐tie groups (87·2 *versus* 89·4 per cent respectively; *P* = 0·386). Neither did the 5‐year relapse‐free survival rate: 76·3 *versus* 77·6 per cent (*P* = 0·765). Significant differences regarding survival were not detected within stages (*Table* 5).

**Table 5:** Long‐term results

	High tie (*n* = 164)	Low tie (*n* = 160)	*P* *
5‐year overall survival rate (%)			
All stages	87·2	89·4	0·386
Stage 1	94·2	96·3	0·740
Stage 2	87·8	84·6	0·965
Stage 3	88·2	88·6	0·880
Stage 4	28·6	72·9	0·109
5‐year relapse‐free survival rate (%)			
All stages	76·3	77·6	0·765
Stage 1	94·2	86·5	0·187
Stage 2	78·1	79·1	0·856
Stage 3	66·2	72·9	0·314

* Log rank test.

## Discussion

In this study the level of IMA ligation in rectal cancer surgery did not affect the anastomotic leak rate. Other factors, including male sex and distance of the tumour from the anal verge, did have an independent influence on the risk of clinical anastomotic leakage. Although this study was stopped prematurely, it is unlikely that level of ligation of the IMA adds significantly to the risk of anastomotic leakage.

Several studies^11^, ^12^, ^13^ have shown that colonic blood flow is decreased in high‐tie compared with low‐tie ligation. Two reports^28^ ^29^ have described the development of proximal bowel necrosis or ischaemia after high‐tie ligation (2 and 0·8 per cent respectively). A haemorrhage test was performed in the present study, but blood flow was not evaluated in a quantitative manner. Differences were not observed between the groups for nearly all short‐term results. The longer IMA tie time in the low‐tie group probably reflects the technical complexity involved in preservation of the LCA. This did not significantly increase total operating time. Some studies^30^ ^31^ have reported that an IMA branching pattern with a large distance between the origins of the IMA and LCA causes technical difficulty. It would appear that surgeons should not hesitate to change the tie level of the IMA when performing a difficult low‐tie ligation.

No significant differences in long‐term results were detected between the two groups, suggesting that low tie with lymph node dissection around the IMA has validity as a surgical treatment. The numbers of lymph nodes harvested around the IMA root and total lymph nodes were not significantly different between groups, in agreement with previous reports^3^ ^4^. Thus both approaches appear equal from an oncological perspective.

This single‐centre study has several limitations. It was stopped prematurely because of slow accrual. This may have introduced bias, although it is unlikely that inclusion of the number of patients assumed in the power calculations would have changed the most important results as the differences between groups were small. Functional evaluations, such as defaecation, digestive symptoms, bladder and sexual functions, were not performed. Lange and colleagues^19^ recommended low‐tie ligation because it allows preservation of the autonomous innervation of the proximal colon. Another study^32^, however, found no difference in defaecatory function or postoperative complications in a relatively small randomized trial. Shiomi and co‐workers^33^ reported that the incidence of anastomotic leakage was lower for low than for high tie in a prospective multicentre cohort study. A randomized multicentre study^34^ is currently in progress. Finally, patients and treatment schedules may differ between Japan and countries in the West, where a significant proportion of patients would have had neodjuvant radiotherapy or chemoradiotherapy, unlike patients in the present study.
